# Assessment of aortic and mitral valve regurgitation volumes by cardiovascular magnetic resonance in participants without valvular heart disease in the Hamburg City Health Study population

**DOI:** 10.1007/s10554-025-03538-z

**Published:** 2025-10-09

**Authors:** Katharina A. Riedl, Georgios Koliopanos, Jan N. Albrecht, Ersin Cavus, Jan-Per Wenzel, Martin Sinn, Bjoern P. Schoennagel, Gunnar K. Lund, Gerhard Adam, Paulus Kirchhof, Stefan Blankenberg, Andreas Ziegler, Kai Muellerleile

**Affiliations:** 1 Department of Cardiology, University Heart and Vascular Center, University Medical Center Hamburg-Eppendorf, Hamburg, Germany; 2 German Center for Cardiovascular Research (DZHK), Partner Site Hamburg/Kiel/Lübeck, Hamburg, Germany; 3 Cardio-CARE, Medizincampus Davos, Davos, Switzerland; 4 Centre for Population Health Innovation (POINT), University Heart and Vascular Center Hamburg, University Medical Center Hamburg-Eppendorf, Hamburg, Germany; 5 School of Mathematics, Statistics and Computer Science, University of KwaZulu-Natal, Pietermaritzburg, South Africa; 6 Department of Diagnostic and Interventional Radiology and Nuclear Medicine, University Medical Center Hamburg-Eppendorf, Hamburg, Germany

**Keywords:** Cardiovascular magnetic resonance, Aortic regurgitation, Mitral regurgitation, Echocardiography, Flow CMR

## Abstract

An assessment of currently available cardiovascular magnetic resonance (CMR) methods to quantify aortic (AR) and mitral regurgitation (MR) in a large, population-based setting is lacking. The aim of this study was to evaluate CMR-based AR and MR quantification methods by quantifying AR and MR in participants without valvular heart disease in the population-based Hamburg City Health Study (HCHS). CMR was performed at 3T in the HCHS population. Aortic valve regurgitation volume (CMR-ARV) was directly obtained from 2D transaortic flow measurements. Mitral valve regurgitation volumes (CMR-MRV) were calculated by subtracting aortic forward flow volumes by Flow CMR from left ventricular stroke volumes by Cine CMR as currently recommended and by a method to account for potential confounding effects of AR on MR calculations (CMR-MRV_MOD_). Absence of AR and MR were assessed by echocardiography as reference technique. The final CMR study population consisted of 1,874 participants (1,261 without any AR, 1,028 without any MR). Median age of the final study population was 66.0 [58.0, 71.0] years, and 41.1% (*n* = 770) of participants were female. Median CMR-ARV was 0.7 [0.2, 1.7] ml in participants without any AR. Median CMR-MRV was 9.4 [4.0, 17.0] ml and median CMR-MRV_MOD_ was 8.7 [4.0, 15.2] ml in participants without any MR, as defined by echocardiography. Unlike accurate, direct ARV measurements by Flow CMR, the currently recommended CMR approach for MRV quantification seems to be affected by a systematic error. MRV measurements by CMR require cautious interpretation, taking into account site specific features.

**Supplementary Information:**

The online version contains supplementary material available at 10.1007/s10554-025-03538-z.

## Introduction

 Aortic (AR) and mitral (MR) valve regurgitation are associated with increased morbidity and mortality [1]. Although echocardiography is the standard technique for assessing AR and MR [2], the quantification of valvular regurgitation is challenging due to the variability and complex geometry of the regurgitation jet [3]. Cine CMR is the reference technique for the assessment of left ventricular (LV) volumes, mass, and function, whereas velocity-encoded (VENC) cardiovascular magnetic resonance enables direct measurements of blood flow volumes (Flow CMR) [4, 5]. Aortic regurgitation volumes (ARV) and fractions (ARF) can be obtained directly by Flow CMR measurements of transaortic flow and are recommended when echocardiography yields ambiguous results [6]. In contrast, quantification of MR by CMR requires a combination of Flow CMR and Cine CMR by subtracting forward flow across the aortic valve by Flow CMR from LV stroke volume (LVSV) by Cine CMR [5]. However, current methods of AR and MR quantification by CMR were originally established in small and predominantly diseased study populations [7–9]. The aim of this study thus was to evaluate current methods for quantification of AR and MR by CMR in a large sample from the population-based Hamburg City Health Study (HCHS) [10].

## Materials and methods

### Study population and design

**Fig. 1:**
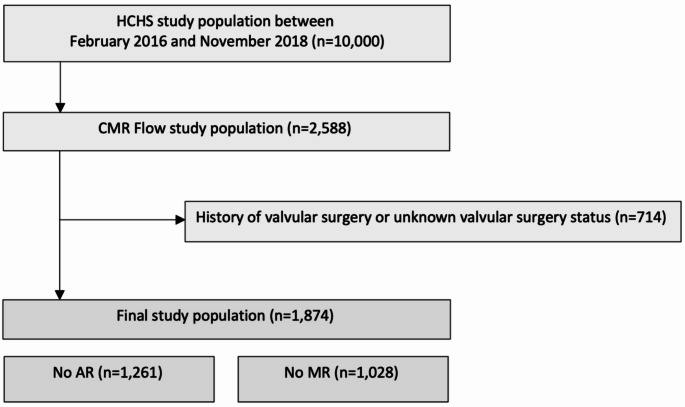
Study flow chart. Flow CMR was performed in 2588 participants of the first 10000 participants of the Hamburg City Health Study. Participants after valve surgery or with unclear valvular surgery status (*n* = 714) were excluded. The remaining participants with native valve status without any MR or AR (*n* = 1874) were defined as the final study population and separated into a subcohort without any AR by echocardiography (*n* = 1261) and a subcohort without any MR by echocardiography (*n* = 1028). HCHS = Hamburg City Health Study, CMR = cardiovascular magnetic resonance, AR = aortic regurgitation, MR = mitral regurgitation.

The Hamburg City Health Study (HCHS) is a single-center, prospective, long-term, population-based, epidemiological cohort study, its rationale and design have been described elsewhere [10]. The present analysis is based on the subset of the first 10000 participants of the HCHS who underwent CMR (2588 participants). Participants between 45 and 74 years were included between February 8, 2016, and November 7, 2018. All participants underwent additionally standard echocardiography, including a qualitative assessment of AR and/or MR [11]. Participants with a history of valvular surgery or unclear valvular surgery status (missing data) and participants with any AR or MR by TTE were excluded from our analysis (Fig. 1).

### Echocardiography

At the baseline visit, all participants of the HCHS underwent a transthoracic echocardiography (TTE) scan using dedicated ultrasound machines (Siemens Acuson SC2000 Prime, Siemens Healthineers) with grey-scale second-harmonic imaging technique at the University Medical Center Hamburg-Eppendorf, Hamburg, Germany. TTE images were acquired following a pre-defined protocol, including all standard echocardiography views and Doppler velocimetry [12]. All scans were recorded as DICOM files and included three consecutive heartbeats.

### CMR protocol

All CMR scans were acquired using a 3 T magnetic resonance imaging (MRI) scanner (MAGNETOM™ Skyra, Siemens Healthineers) at the University Medical Center Hamburg-Eppendorf, Hamburg, Germany, following a protocol published elsewhere [13]. In brief, the CMR protocol included through-plane 2D phase contrast (PC) Flow CMR flow measurements at the tip of the aortic valve leaflets and standard Cine CMR short- and long-axis measurements [13, 14]. Typical Flow CMR parameters were: repetition time (TR) 4.3 ms, echo time (TE) 2.3 ms, flip angle (FA) 20°, field of view (FoV) 340 mm^2^, voxel size 1.8 × 1.8 × 6 mm^3^, parallel acquisition factor (PAT) 2. Typical Cine CMR (bSSFP) parameters were: TR 3.3 ms, TE 1.5 ms, FA 80°, FoV 340 mm^2^, voxel size 1.6 × 1.6 × 8 mm^3^.

### CMR data analysis

**Fig. 2:**
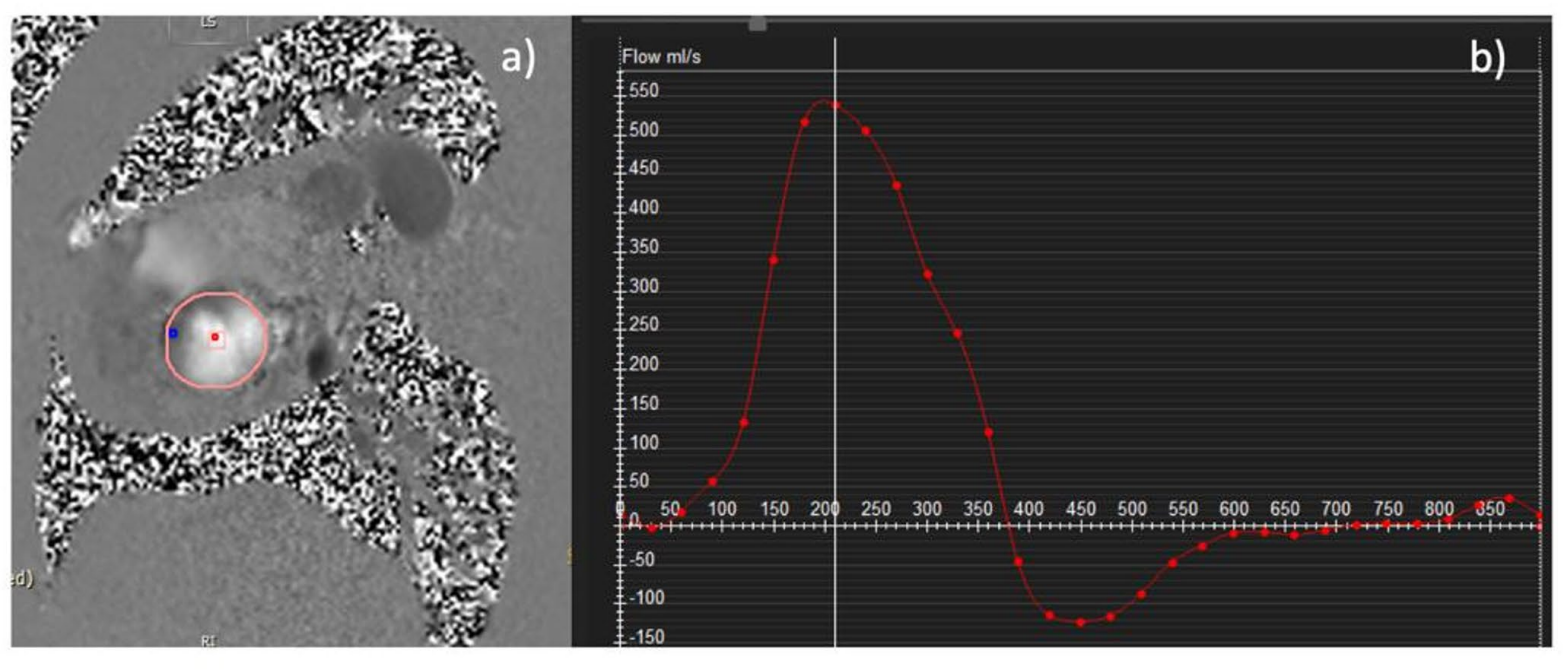
Example Aortic Flow CMR. 2D phase contrast CMR scan **(a)** and generated aortic flow curve with flow [ml/s] over time **(b)**. CMR = cardiovascular magnetic resonance

Raw data were transferred to the dedicated cvi42 (Circle Cardiovascular Imaging Inc.) software, version 5.6.6, for CMR data analysis according to a standardized protocol. Every tenth CMR data set was analyzed by a second observer, who was blinded to the findings of the first observer, enabling the assessment of inter-observer agreement. Through-plane Flow CMR measurements were obtained for the aortic valve by placing the region of interest (ROI) at the tip of the aortic valve cusps and directly measuring the aortic forward (AFV_Flow CMR_) and backward flow volumes (ABV_Flow CMR_) as recommended (Fig. 2) [15]. For flow analysis, a background correction ROI was placed in the skeletal muscle in the magnitude images. LV segmentation was performed by cvi42, version 5.6.6, using a semi-automated threshold-based method with manual refinement [13]. LV end-diastolic (LVEDV) and end-systolic (LVESV) volumes were obtained from short axis Cine CMR stacks from base to apex including the papillary muscles into the LV mass to calculate LV stroke volumes (LVSV_Cine CMR_) [5, 13, 16].

The Flow CMR method to measure CMR-AR was based on direct measurements of transvalvular flow [16, 17]:

$$CMR\text{-}ARV\left[ml\right]={ABV}_{Flow\:CMR}\left[ml\right],$$        

$$\begin{aligned}&CMR\text{-}ARF\left[\%\right]\\&\quad=\left(\raisebox{1ex}{${ABV}_{Flow\:CMR}\left[ml\right]$}\!\left/\:\!\raisebox{-1ex}{${AFV}_{Flow\:CMR}\left[ml\right]$}\right.\right)x\:100\%,\end{aligned}$$  

where CMR denotes cardiovascular magnetic resonance, ARV is the aortic regurgitation volume, ABV_Flow CMR_ the aortic backward flow volume via Flow CMR, ARF the aortic regurgitation fraction, and AFV_Flow CMR_ the aortic forward flow volume via Flow CMR.

We calculated CMR-MR by subtracting aortic forward flow volume by Flow CMR from LV stroke volume by Cine CMR [18]:

$$\begin{aligned}\:&CMR\text{-}MRV\left[ml\right]\\&\quad={LVSV}_{Cine\:CMR}\left[ml\right]-{AFV}_{Flow\:CMR}\left[ml\right],\end{aligned}$$

$$\begin{aligned}&CMR\text{-}MRF\left[\%\right]\\&\quad=\left(\raisebox{1ex}{$CMR\text{-}MRV\left[ml\right]$}\!\left/\:\!\raisebox{-1ex}{${LVSV}_{Cine\:CMR}\left[ml\right]$}\right.\right)x\:100\%,\end{aligned}$$

with CMR denoting the cardiovascular magnetic resonance, MRV is the mitral regurgitation volume, LVSV_Cine CMR_ the left ventricular stroke volume via Cine CMR, AFV_Flow CMR_ the aortic forward flow volume via Flow CMR, and MRF the mitral regurgitation fraction.

CMR-MR was also estimated by subtracting the total of ABV_Flow CMR_ and AFV_Flow CMR_ from the LV stroke volume by Cine CMR to account for the potential confounding effect of AR on MR calculations [19]:

$$\begin{aligned}&{CMR\text{-}MRV}_{MOD}\left[ml\right]={LVSV}_{Cine\:CMR}\left[ml\right]\\&\quad-\left({ABV}_{Flow\:CMR}\left[ml\right]+{AFV}_{Flow\:CMR}\left[ml\right]\right),\end{aligned}$$

$$\begin{aligned}&{CMR\text{-}MRF}_{MOD}\left[\%\right]\\&\quad=\left(\raisebox{1ex}{${CMR\text{-}MRV}_{MOD}\left[ml\right]$}\!\left/\:\!\raisebox{-1ex}{${LVSV}_{Cine\:CMR}\left[ml\right]$}\right.\right)x\:100\%,\end{aligned}$$

where CMR is the cardiovascular magnetic resonance, MRV_MOD_ the mitral regurgitation volume considering possible AR, LVSV_Cine CMR_ the left ventricular stroke volume via Cine CMR, ABV_Flow CMR_ the aortic backward flow volume via Flow CMR, AFV_Flow CMR_ the aortic forward volume via Flow CMR, and MRF_MOD_ the mitral regurgitation fraction considering possible AR.

### Statistical analysis

Continuous data are presented as median and quartiles. Categorical data are presented as absolute numbers with percentages. Bland-Altman analyses were performed to assess interobserver-agreements [20]. Differences according to sex and age were assessed by comparing ARV and MRV between participants ≤65 years vs. >65 years of age, as well as between females and males by a Mann-Whitney U test [21]. All statistical analyses were performed in R version 4.3.0.

## Results

The final CMR study population consisted of 1874 participants, including 1261 participants without any AR and 1028 participants without any MR after the exclusion of 714 participants with a history of valvular surgery and with unknown valvular surgery status (Fig. 1). The median age of the final study population (*n* = 1874) was 66.0 [58.0, 71.0] years, and 41.1% (*n* = 770) of participants were female. The study population was thus older and had a lower proportion of female participants compared to the general HCHS population (Table 1).

**Table 1:** Demographics and clinical characteristics

	First 10000 participants of the HCHS (*n* = 10000)	CMR Flow study population (*n* = 2588)	Final study population (*n* = 1874)
Age (years)	63.0 [55.0, 70.0]	67.0 [59.0, 72.0]	66.0 [58.0, 71.0]
Female sex	5108 (51.1)	1071 (41.4)	770 (41.1)
History of valvular surgery	576 (7.7)	145 (7.2)	0 (0.0)
Current or history of smoking	6384 (64.2)	1691 (65.5)	1197 (64.0)
Diabetes mellitus	794 (8.6)	247 (10.1)	163 (9.3)
BMI (kg/m^2^)	26.1 [23.5, 29.2]	26.5 [23.9, 29.4]	26.3 [23.8, 29.3]
NT-proBNP (pg/ml)	81.0 [44.0, 148.0]	86.0 [47.0, 161.0]	82.0 [45.0, 155.0]
LVEDMi (ml)	62.3 [53.8, 72.2]	62.3 [53.8, 72.2]	62.0 [53.5, 72.2]
LVEDVi (ml)	62.1 [52.1, 71.7]	62.1 [52.1, 71.7]	62.4 [52.4, 72.1]
LVESVi (ml)	18.6 [14.1, 23.7]	18.6 [14.1, 23.7]	18.8 [14.3, 23.7]
LVSVi (ml)	42.8 [36.0, 49.5]	42.8 [36.0, 49.5]	43.0 [36.1, 49.7]
LVEF (%)	69.6 [64.0, 74.9]	69.6 [64.0, 74.9]	69.3 [64.0, 74.7]

Continuous data are median and interquartile range. Categorial data are absolute numbers with percentages. HCHS = Hamburg City Heath Study, CMR = cardiovascular magnetic resonance, BMI = Body-mass index, NT-proBNP = N-terminal pro Brain Natriuretic Peptid, LVEDMi = left ventricular enddiastolic myocardial mass index, LVEDVi = left ventricular enddiastolic volume index, LVESVi = left ventricular endsystolic volume index, LVSV = left ventricular stroke volume index, LVEF = left ventricular ejection fraction

### Interobserver agreement

Mean interobserver differences were 0.005 ± 0.033 ml for ARV and 0.789 ± 9.614 for MRV measurements, respectively (Supplemental Table 1).

### Regurgitation volumes and fractions

**Fig. 3:**
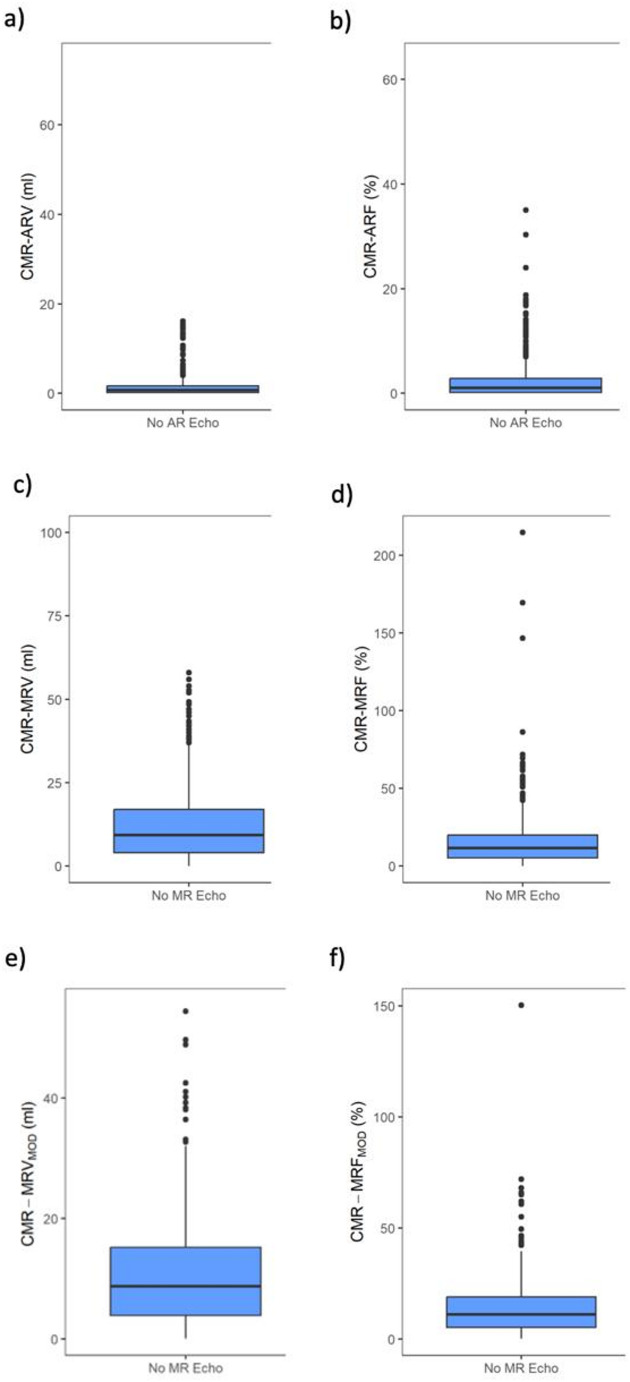
AR and MR regurgitation volumes and fractions by CMR. Boxplots for CMR-ARV **(a)**, CMR-ARF **(b)**, CMR-MRV **(c)**, CMR-MRF **(d)**, CMR-MRV_MOD_ **(e)** and CMR-MRF_MOD_ **(f)**. CMR = cardiovascular magnetic resonance, ARV = aortic regurgitation volume, ARF = aortic regurgitation fraction, AR = aortic regurgitation, MRV = mitral regurgitation volume, MRF = mitral regurgitation fraction, MR = mitral regurgitation, MRV_MOD_ = mitral regurgitation volume, considering possible AR, MRF_MOD_ = mitral regurgitation fraction, considering possible AR.

Median CMR-ARV (CMR-ARF) was 0.7 [0.2, 1.7] ml (1.0% [0.2%, 2.8%]) in participants without any AR by echocardiography (Table 2; Fig. 3a, b). Median CMR-MRV (CMR-MRF) was 9.4 [4.0, 17.0] ml (11.7% [5.3%, 20.0%]) in participants without any MR by echocardiography (Table 2; Fig. 3c, d). Results were similar for the modified approach, considering the potential confounding effects of coincident AR on MR calculations with median CMR-MRV_MOD_ (CMR-MRF_MOD_) 8.7 [4.0, 15.2] ml (11.1% [5.3%, 19.0%]) (Table 2; Fig. 3e, f).

**Table 2:** AR and MR regurgitation volumes by CMR in participants without valvular heart disease as defined by echocardiography

*Participants without any AR*	*n = 1261*
CMR-ARV (ml)	0.7 [0.2, 1.7]
CMR-ARF (%)	1.0 [0.2, 2.8]
*Participants without any MR*	*n = 1028*
CMR-MRV (ml)	9.4 [4.0, 17.0]
CMR-MRF (%)	11.7 [5.3, 20.0]
CMR-MRV_MOD_ (ml)	8.7 [4.0, 15.2]
CMR-MRF_MOD_ (%)	11.1 [5.3, 19.0]

Categorial data are absolute numbers with percentages. Correlation is polyserial correlation. AR = aortic regurgitation, CMR = cardiovascular magnetic resonance, ARV = aortic regurgitation volume = backward volume, ARF = aortic regurgitation fraction, MR = mitral regurgitation, MRV = mitral regurgitation volume, MRF = mitral regurgitation fraction, MRV_MOD_ = mitral regurgitation volume, considering possible AR, MRF_MOD_ = mitral regurgitation fraction, considering possible AR

### Regurgitation volumes and fractions according to age and sex categories

Median CMR-ARV was significantly higher in participants ≤65 years compared with > 65 years (0.01 [0.00, 0.02] vs. 0.01 [0.00, 0.03], *p* = 0.006; Supplemental Table 2). Median CMR-MRV, CMR-MRF, CMR-MRV_MOD_ and CMR-MRF_MOD_ were not significantly different between both age groups (*p* = 0.130, *p* = 0.880, *p* = 0.370 and *p* = 0.575; Supplemental Table 2). Median CMR-ARV, CMR-ARF, CMR-MRV, CMR-MRF and CMR-MRV_MOD_ were significantly higher in males compared to females (*p* < 0.001, *p* < 0.001, *p* < 0.001, *p* = 0.011 and *p* < 0.001; Supplemental Table 3).

## Discussion

This study quantified AR and MR volume measurements by CMR in valve-healthy participants without any AR or MR by echocardiography in the population-based HCHS cohort. The three major findings were:

- We did not find a systematic error in direct ARV measurements by Flow CMR.
- There was a systematic overestimation of MRV measurements by CMR, using the difference in LV stroke volumes between Cine CMR and Flow CMR, as currently recommended.
- A modified approach, considering the potential confounding effects of coincident AR on MR calculations, provided a similar, systematic overestimation of MRV measurements by CMR.

### Aortic valve regurgitation volumes by CMR in healthy individuals

**Fig. 4:**
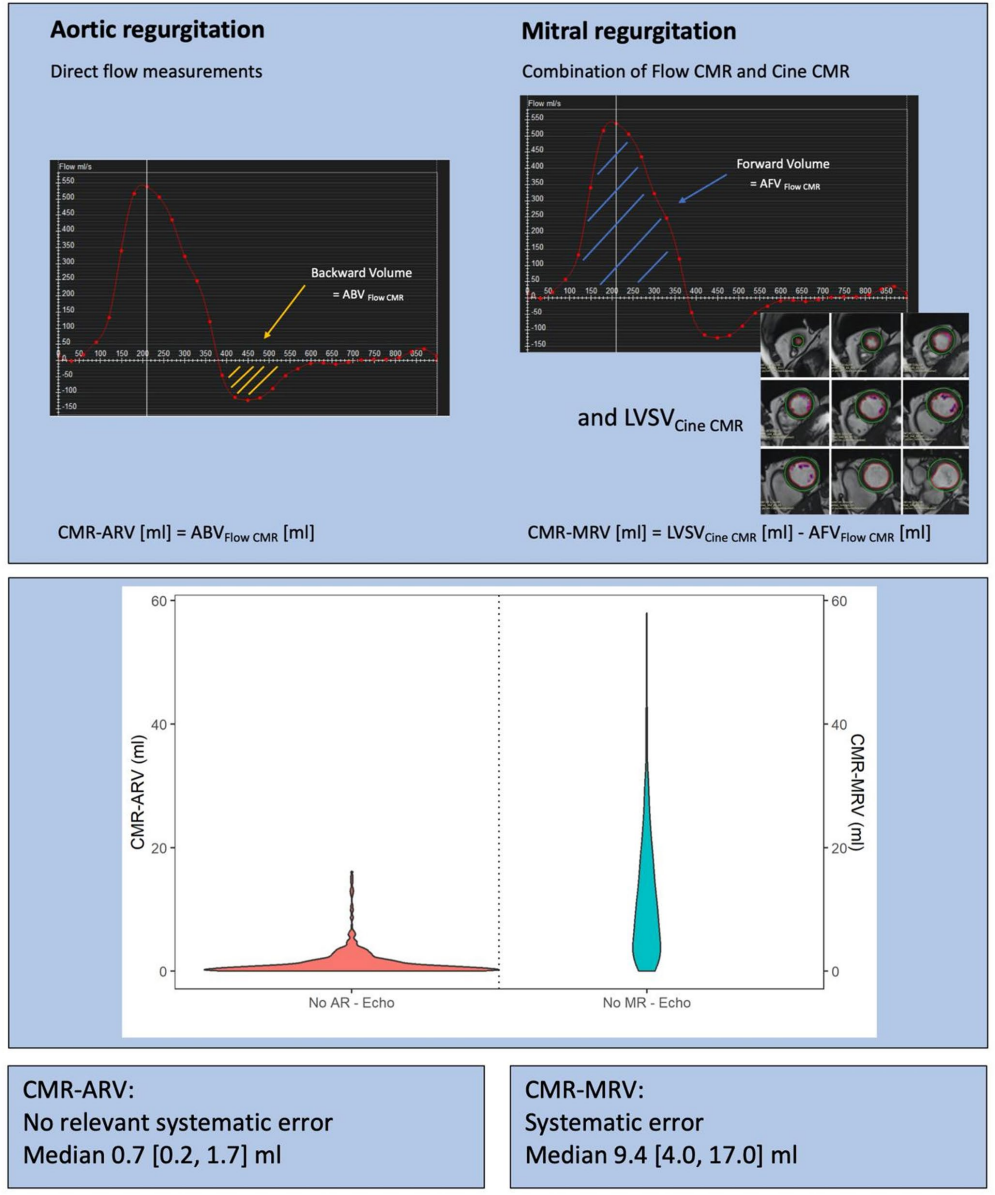
Graphical Abstract. CMR-based Quantification of AR and MR in valve-healthy participants in the HCHS. CMR = cardiovascular magnetic resonance, ARV = aortic regurgitation volume, ABV_Flow CMR_ = aortic backward flow volume via Flow CMR, LVSV_Cine CMR_ = left ventricular stroke volume via Cine CMR, MRV = mitral regurgitation volume, AFV_Flow CMR_ = aortic forward volume via Flow CMR, HCHS = Hamburg City Health Study.

Direct ARV measurements by Flow CMR offer the advantages of fast data acquisition within a single breath-hold and a simple data analysis [5]. This study includes, by far, the largest population with Flow CMR data in “healthy” subjects without echocardiographic evidence for AR. We observed negligible median ARV and ARF of 0.7 ml and 1.0% by CMR in these individuals, respectively (Table 2; Fig. 3). In addition, we found a strong interobserver agreement for ARV measurements (Supplemental Table 1). Our findings surpass a recent report by Gabriel et al. who observed a mean CMR-ARV of 4.0 ml (4.5%) in 46 individuals without any AR by echocardiography with a similar technical approach [15] and by Gelfand et al. who reported a mean CMR-ARF of 2 ± 2% in 35 subjects without any AR by echocardiography [9]. Our findings underscore the absence of a systematic error in these simple, direct AR measurements by Flow CMR (Fig. 4).

### Mitral valve regurgitation volumes by CMR in healthy individuals

Unlike the simple approach for ARV measurements, the quantification of MRV by CMR is based on the difference between aortic forward flow volumes by Flow CMR and LV stroke volumes by Cine CMR [18]. However, the combination of two different CMR modalities with data acquisition from different heartbeats inherently comes along with the risk of systematic errors. We found a relevant median MRV of 9.4 ml in “healthy” individuals without MR by echocardiography, which needs to be interpreted as the systematic, “baseline error” of the CMR method. Current literature on this topic is scarce: Kizilbash et al. reported CMR-MRV measurements in four “healthy” individuals without any MR and found a mean CMR-MRV of 10 ± 5 ml [22]. Gelfand et al. reported a CMR-MRF of 10 ± 9% in 35 individuals without echocardiographic evidence for MR [9]. In agreement with our findings, Altes et al. also demonstrated significantly higher CMR-based MRV and MRF values in males compared to females, reflecting the generally larger LV volumes in male individuals [23]. These numbers agree perfectly with our findings and point out the insufficient validation of this method, questioning the current perception of CMR as a method with incremental value for assessing the severity of MR. This finding is of immediate clinical importance since measurements of MRV and/or MRF by CMR could result in incorrectly “upgrading” the severity of MR by CMR [6].

Although the lack of a true “gold standard” for assessing valvular regurgitation volumes prevents a definite proof, this “baseline error” could theoretically be related to two different reasons: On the one hand, CMR measurements could have revealed a systematic underestimation of mild mitral valve regurgitation by echocardiography in our population. However, this explanation is very unlikely in our large, population-based cohort and would assume the presence of significant MR in the vast majority of the general population. On the other hand, and much more likely, there seems to be a systematic overestimation of MRV by CMR. In particular, LV volumetry by Cine CMR is affected by some pitfalls, such as the inclusion of the papillary muscles, which affects the calculation of LV stroke volumes [24]. We included the papillary muscles to the LV mass, which is “biologically” correct and results in lower LV stroke volumes compared to the alternative approach of including the papillary muscles into LV volumes [25]. Therefore, it is unlikely that our approach to the papillary muscles was the reason for an overestimation of LV stroke volumes by Cine CMR. However, consistent contouring of the papillary muscles in end-diastole and end-systole is challenging and could have contributed to our observation [25]. Furthermore, we applied two different methods for calculating CMR-MRV, in order to rule out a potential confounding effect of aortic regurgitation on the estimation of MRV by CMR [19], but there was only a marginal difference between the median MRV and MRF values between both methods in our population including a low number of individuals with AR. Independent from the potential underlying reasons, our findings clearly reveal a significant “baseline” error of MRV quantification by CMR (Fig. 4), which has been, albeit reported earlier, widely neglected so far [9, 22]. Future studies addressing this topic should include transesophageal echocardiography, TTE and CMR at the same day, including healthy individuals, but also diseased patients with a spectrum of all MR severity levels.

### Limitations

The absence of a true quantitative “gold standard”, particularly for assessing MR, constitutes a principal limitation of all studies on valvular regurgitation. Direct MR measurements by CMR are confounded by LVOT flow, but also by the movement of the mitral valve annulus through the slice itself, both affecting VENC CMR based flow measurements. Of note, the CMR method to quantify MRV depends on accurate aortic flow measurements as well as on LV volumetry. In this context, it is important to note that CMR flow measurements, and our findings, are confined to the specific setting, including vendor, field strength, post-processing software, but also even the individual machine, which could limit the generalizability of our findings. A further evaluation using flow phantoms could validate the accuracy of flow measurements of our specific setting and confirm our findings. Moreover, due to the population-based setting of the HCHS, TTE and CMR were not performed at the same day [26], which could represent a potential bias. However, this analysis included only participants without AR and MR by TTE and it is very unlikely that the temporal interval between both scans could have resulted in relevant underestimation and/or missing MR by TTE in such a large population. Additional potential limitations include potential effects of the order of flow measurements and Cine CMR, as well as of missing information on shunts on MRV estimations. However, the amount of these potential errors appears to be neglectable, given the short interval between flow measurements and Cine CMR, as well as the low prevalence of shunts in the population [27]. In the HCHS, we used version 5.6.6 of the cvi42 software with a semi-automated threshold-based method with manual refinement and the study design of the HCHS does not allow the use of updated software versions in order to guarantee data stability. Therefore, the impact of novel AI-based methods on AR and MR quantification by tools needs to be addressed in future studies. Nevertheless, this study is unrivaled regarding its sample size and also by the highly standardized performance of Flow CMR and echocardiography to assess valvular regurgitation volumes in a population setting [19].

## Conclusion

Our findings indicate that Flow CMR can assess ARV without a relevant systematic error. In contrast, the currently recommended CMR approach seems to be affected by a systematic overestimation of MRV. Our results call for a critical appraisal of the role of CMR to quantify mitral regurgitation, considering site specific features, such as vendor, field strength and post-processing software.

## Supplementary Information

Below is the link to the electronic supplementary material.

Supplementary Material 1

## Data Availability

The data underlying this article will be shared on reasonable request to the corresponding author.

## Abbreviations

AR: Aortic regurgitation
ARF: Aortic regurgitation fraction
ARV: Aortic regurgitation volume
CMR: Cardiovascular magnetic resonance
Cvi42: Circle Cardiovascular Imaging Inc
HCHS: Hamburg City Health Study
LVEDVi: Left ventricular end-diastolic volume index
LVEDMi: Left ventricular end-diastolic myocardial mass index
LVEF: Left ventricular ejection fraction
LVESVi: Left ventricular end-systolic volume index
LV: Left ventricular/ventricle
LVSF: Left ventricular systolic function
LVSV: Left ventricular stroke volume
LVSVi: Left ventricular stroke volume index
MR: Mitral regurgitation
MRF: Mitral regurgitation fraction
MRF_Mod_: Mitral regurgitation fraction, considering possible AR
MRV: Mitral regurgitation volume
MRV_Mod_: Mitral regurgitation volume, considering possible AR
PC: Phase Contrast
ROI: Region of interest
VENC: Velocity-encoded

## References

[1] d’Arcy JL, Coffey S, Loudon MA, Kennedy A, Pearson-Stuttard J et al (2016) Large-scale community echocardiographic screening reveals a major burden of undiagnosed valvular heart disease in older people: the oxvalve population cohort study. Eur Heart J 37:3515–3522. 10.1093/eurheartj/ehw229 27354049 10.1093/eurheartj/ehw229 PMC5216199

[2] Lancellotti P, Tribouilloy C, Hagendorff A, Popescu BA, Edvardsen T et al (2013) Recommendations for the echocardiographic assessment of native valvular regurgitation: an executive summary from the European association of cardiovascular imaging. Eur Heart J Cardiovasc Imaging 14:611–644. 10.1093/ehjci/jet105 23733442 10.1093/ehjci/jet105

[3] Iung B, Delgado V, Rosenhek R, Price S, Prendergast B et al (2019) Contemporary presentation and management of valvular heart disease: the eurobservational research programme valvular heart disease II survey. Circulation 140:1156–1169. 10.1161/circulationaha.119.041080 31510787 10.1161/CIRCULATIONAHA.119.041080

[4] Nishimura RA, Otto CM, Bonow RO, Carabello BA, Erwin JP 3rd, et al (2017) 2017 AHA/ACC focused update of the 2014 AHA/ACC guideline for the management of patients with valvular heart disease: A report of the American college of Cardiology/American heart association task force on clinical practice guidelines. J Am Coll Cardiol 70:252–289. 10.1016/j.jacc.2017.03.011 28315732 10.1016/j.jacc.2017.03.011

[5] Schulz-Menger J, Bluemke DA, Bremerich J, Flamm SD, Fogel MA et al (2020) Standardized image interpretation and post-processing in cardiovascular magnetic resonance – 2020 update: society for cardiovascular magnetic resonance (SCMR): board of trustees task force on standardized Post-Processing. J Cardiovasc Magn Reson 22:19. 10.1186/s12968-020-00610-6 32160925 10.1186/s12968-020-00610-6 PMC7066763

[6] Vahanian A, Beyersdorf F, Praz F, Milojevic M, Baldus S et al (2022) 2021 ESC/EACTS guidelines for the management of valvular heart disease. Eur Heart J 43:561–632. 10.1093/eurheartj/ehab395 34453165 10.1093/eurheartj/ehab395

[7] Globits S, Frank H, Mayr H, Neuhold A, Glogar D (1992) Quantitative assessment of aortic regurgitation by magnetic resonance imaging. Eur Heart J 13:78–83. 10.1093/oxfordjournals.eurheartj.a060052 1577036 10.1093/oxfordjournals.eurheartj.a060052

[8] Kozerke S, Schwitter J, Pedersen EM, Boesiger P (2001) Aortic and mitral regurgitation: quantification using moving slice velocity mapping. J Magn Reson Imaging 14:106–112. 10.1002/jmri.1159 11477667 10.1002/jmri.1159

[9] Gelfand EV, Hughes S, Hauser TH, Yeon SB, Goepfert L et al (2006) Severity of mitral and aortic regurgitation as assessed by cardiovascular magnetic resonance: optimizing correlation with doppler echocardiography. J Cardiovasc Magn Reson 8:503–507. 10.1080/10976640600604856 16755839 10.1080/10976640600604856

[10] Jagodzinski A, Johansen C, Koch-Gromus U, Aarabi G, Adam G et al (2020) Rationale and design of the Hamburg City health study. Eur J Epidemiol 35:169–181. 10.1007/s10654-019-00577-4 31705407 10.1007/s10654-019-00577-4 PMC7125064

[11] Vahanian A, Beyersdorf F, Praz F, Milojevic M, Baldus S et al (2022) 2021 ESC/EACTS guidelines for the management of valvular heart disease: developed by the task force for the management of valvular heart disease of the European society of cardiology (ESC) and the European association for Cardio-Thoracic surgery (EACTS). Revista Española de Cardiología (English Edition). 10.1016/j.rec.2022.05.006 10.1016/j.rec.2022.05.006 35636831

[12] Wenzel JP, Petersen E, Nikorowitsch J, Senftinger J, Sinning C et al (2021) Transthoracic echocardiographic reference values of the aortic root: results from the Hamburg City health study. Int J Cardiovasc Imaging 37:3513–3524. 10.1007/s10554-021-02354-5 34324091 10.1007/s10554-021-02354-5 PMC8604854

[13] Bohnen S, Avanesov M, Jagodzinski A, Schnabel RB, Zeller T et al (2018) Cardiovascular magnetic resonance imaging in the prospective, population-based, Hamburg City health cohort study: objectives and design. J Cardiovasc Magn Reson 20:68. 10.1186/s12968-018-0490-7 30244673 10.1186/s12968-018-0490-7 PMC6151919

[14] Westenberg JJ, Roes SD, Ajmone Marsan N, Binnendijk NM, Doornbos J et al (2008) Mitral valve and tricuspid valve blood flow: accurate quantification with 3D velocity-encoded MR imaging with retrospective valve tracking. Radiology 249:792–800. 10.1148/radiol.2492080146 18849503 10.1148/radiol.2492080146

[15] Gabriel RS, Renapurkar R, Bolen MA, Verhaert D, Leiber M et al (2011) Comparison of severity of aortic regurgitation by cardiovascular magnetic resonance versus transthoracic echocardiography. Am J Cardiol 108:1014–1020. 10.1016/j.amjcard.2011.05.034 21784393 10.1016/j.amjcard.2011.05.034

[16] Lee JC, Branch KR, Hamilton-Craig C, Krieger EV (2018) Evaluation of aortic regurgitation with cardiac magnetic resonance imaging: a systematic review. Heart 104:103–110. 10.1136/heartjnl-2016-310819 28822982 10.1136/heartjnl-2016-310819

[17] Mathew RC, Löffler AI, Salerno M (2018) Role of cardiac magnetic resonance imaging in valvular heart disease: diagnosis, assessment, and management. Curr Cardiol Rep 20:119. 10.1007/s11886-018-1057-9 30259253 10.1007/s11886-018-1057-9 PMC6415765

[18] Garg P, Swift AJ, Zhong L, Carlhäll CJ, Ebbers T et al (2020) Assessment of mitral valve regurgitation by cardiovascular magnetic resonance imaging. Nat Rev Cardiol 17:298–312. 10.1038/s41569-019-0305-z 31819230 10.1038/s41569-019-0305-z PMC7165127

[19] Uretsky S, Argulian E, Narula J, Wolff SD (2018) Use of cardiac magnetic resonance imaging in assessing mitral regurgitation: current evidence. J Am Coll Cardiol 71:547–563. 10.1016/j.jacc.2017.12.009 29406861 10.1016/j.jacc.2017.12.009

[20] Bland JM, Altman DG (1986) Statistical methods for assessing agreement between two methods of clinical measurement. Lancet 1:307–310 2868172

[21] Nachar N (2008) The mann-Whitney U: a test for assessing whether two independent samples come from the same distribution. Tutorials Quant Methods Psychol. 10.20982/tqmp.04.1.p013

[22] Kizilbash AM, Hundley WG, Willett DL, Franco F, Peshock RM et al (1998) Comparison of quantitative doppler with magnetic resonance imaging for assessment of the severity of mitral regurgitation. Am J Cardiol 81:792–795. 10.1016/s0002-9149(97)01024-2 9527098 10.1016/s0002-9149(97)01024-2

[23] Altes A, Levy F, Hanet V, De Azevedo D, Krug P et al (2024) Impact of sex on severity assessment and cardiac remodeling in primary mitral regurgitation. JACC Adv 3:101023. 10.1016/j.jacadv.2024.101023 39130021 10.1016/j.jacadv.2024.101023 PMC11312794

[24] Kawel-Boehm N, Hetzel SJ, Ambale-Venkatesh B, Captur G, Francois CJ et al (2020) Reference ranges (normal values) for cardiovascular magnetic resonance (CMR) in adults and children: 2020 update. J Cardiovasc Magn Reson 22:87. 10.1186/s12968-020-00683-3 33308262 10.1186/s12968-020-00683-3 PMC7734766

[25] Sievers B, Kirchberg S, Bakan A, Franken U, Trappe HJ (2004) Impact of papillary muscles in ventricular volume and ejection fraction assessment by cardiovascular magnetic resonance. J Cardiovasc Magn Reson 6:9–16. 10.1081/jcmr-120027800 15054924 10.1081/jcmr-120027800

[26] Wenzel JP, Albrecht JN, Toprak B, Petersen E, Nikorowitsch J et al (2025) Head-to-head comparison of cardiac magnetic resonance imaging and transthoracic echocardiography in the general population (MATCH). Clin Res Cardiol. 10.1007/s00392-025-02660-1 40353872 10.1007/s00392-025-02660-1

[27] Marelli AJ, Mackie AS, Ionescu-Ittu R, Rahme E, Pilote L (2007) Congenital heart disease in the general population: changing prevalence and age distribution. Circulation 115:163–172. 10.1161/circulationaha.106.627224 17210844 10.1161/CIRCULATIONAHA.106.627224

