# Supplementary material for: Assessment of aortic and mitral valve regurgitation volumes by cardiovascular magnetic resonance in participants without valvular heart disease in the Hamburg City Health Study population
Source: Int J Cardiovasc Imaging. 2025 Oct 9;41(12):2343–52. doi: 10.1007/s10554-025-03538-z (PMC12678579; doi:10.1007/s10554-025-03538-z)
Supplement: Supplementary file 1 — Supplementary Material 1 [file 10554_2025_3538_MOESM1_ESM.docx]

**Supplemental Material**

**Supplemental Table 1 Bland-Altman interobserver agreements analyses of ARV and MRV**

|  | **ARV** | **MRV** |
| --- | --- | --- |
| Mean difference (ml) | 0.005 | 0.789 |
| Standard Deviation of differences (ml) | 0.033 | 9.614 |

*CMR = cardiovascular magnetic resonance, ARV = aortic regurgitation volume, MRV = mitral regurgitation volume.*

**Supplemental Table 2 AR and MR regurgitation volumes and fractions according to age categories**

|  | **Age ≤65 years** | **Age >65 years** | **p-values** |
| --- | --- | --- | --- |
| **AR data** | **n = 662** | **n=599** |  |
| CMR-ARV (ml) | 0.01 [0.00, 0.02] | 0.01 [0.00, 0.03] | 0.006 |
| CMR-ARF (%) | 1.00 [0.05, 2.67] | 1.00 [0.36, 2.89] | 0.070 |
| **MR data** | **n = 560** | **n=468** |  |
| CMR-MRV (ml) | 10.00 [4.53, 17.10] | 9.00 [4.00, 16.00] | 0.130 |
| CMR-MRF (%) | 11.69 [5.44, 19.68] | 11.73 [5.19, 20.59] | 0.880 |
| CMR-MRV_MOD_ (ml) | 9.48 [4.06, 16.73] | 8.02 [3.90, 13.68] | 0.370 |
| CMR-MRF_MOD_ (%) | 10.94 [4.81, 19.03] | 11.35 [5.42, 18.86] | 0.575 |

*CMR = cardiovascular magnetic resonance, AR = aortic regurgitation, MR = mitral regurgitation, ARV = aortic regurgitation volume, ARF = aortic regurgitation fraction, MRV = mitral regurgitation volume, MRF = mitral regurgitation fraction, MRV_MOD_ = mitral regurgitation volume, considering possible AR, MRF_MOD_ = mitral regurgitation fraction, considering possible AR.*

**Supplemental Table 3 AR and MR regurgitation volumes and fractions according to sex**

|  | **Male** | **Female** | **p-values** |
| --- | --- | --- | --- |
| **AR data** | **n = 734** | **n=527** |  |
| CMR-ARV (ml) | 0.01 [0.01, 0.03] | 0.00 [0.00, 0.02] | <0.001 |
| CMR-ARF (%) | 1.44 [0.69, 3.19] | 1.00 [0.00, 2.00] | <0.001 |
| **MR data** | **n = 612** | **n=416** |  |
| CMR-MRV (ml) | 11.59 [4.36, 19.00] | 7.98 [4.00, 14.00] | <0.001 |
| CMR-MRF (%) | 12.61 [5.77, 21.23] | 10.94 [5.19, 18.83] | 0.011 |
| CMR-MRV_MOD_ (ml) | 10.36 [4.69, 17.06] | 7.11 [3.43, 11.70] | <0.001 |
| CMR-MRF_MOD_ (%) | 11.87 [5.27, 20.53] | 9.88 [5.30, 16.99] | 0.070 |

*CMR = cardiovascular magnetic resonance, AR = aortic regurgitation, MR = mitral regurgitation, ARV = aortic regurgitation volume, ARF = aortic regurgitation fraction, MRV = mitral regurgitation volume, MRF = mitral regurgitation fraction, MRV_MOD_ = mitral regurgitation volume, considering possible AR, MRF_MOD_ = mitral regurgitation fraction, considering possible AR.*
